# Pupil Dilation and EEG Alpha Frequency Band Power Reveal Load on Executive Functions for Link-Selection Processes during Text Reading

**DOI:** 10.1371/journal.pone.0130608

**Published:** 2015-06-15

**Authors:** Christian Scharinger, Yvonne Kammerer, Peter Gerjets

**Affiliations:** Knowledge Media Research Center, Tuebingen, Germany; University of Granada, SPAIN

## Abstract

Executive working memory functions play a central role in reading comprehension. In the present research we were interested in additional load imposed on executive functions by link-selection processes during computer-based reading. For obtaining process measures, we used a methodology of concurrent electroencephalographic (EEG) and eye-tracking data recording that allowed us to compare epochs of pure text reading with epochs of hyperlink-like selection processes in an online reading situation. Furthermore, this methodology allowed us to directly compare the two physiological load-measures EEG alpha frequency band power and pupil dilation. We observed increased load on executive functions during hyperlink-like selection processes on both measures in terms of decreased alpha frequency band power and increased pupil dilation. Surprisingly however, the two measures did not correlate. Two additional experiments were conducted that excluded potential perceptual, motor, or structural confounds. In sum, EEG alpha frequency band power and pupil dilation both turned out to be sensitive measures for increased load during hyperlink-like selection processes in online text reading.

## Introduction

Imagine you want to gather some information about a certain topic. You may start by reading an introductory article on 'Wikipedia' (http://en.wikipedia.org/), nowadays the standard online encyclopedia in the Internet. While reading the article you will be confronted with hyperlinks that lead to further web pages with additional information. These hyperlinks may be matching your target topic, or they may be only partly relevant or even irrelevant for your current information gathering process [1]. In all cases the hyperlinks will interrupt your current reading process and will call for additional decision processes, that is, you have to decide which links to follow or which links to ignore [2].

Cognitively, these decision processes induced by hyperlinks can be expected to increase load on executive functions (EFs) that are already loaded during normal reading [3]. EFs can be defined as attention-related top-down control processes that are necessary to accomplish complex cognitive tasks that require adaptive behavior [4]. EFs are usually conceptualized to reside within the central-executive component of working memory [5,6]. Often differentiated core EFs are updating, shifting, and inhibition [7,8]. Although being differentiable, these core EFs have been shown to share a common underlying factor that has been attributed to processes of controlled attention [8]. The EF labeled 'updating' is generally defined to incorporate core processes of working memory (WM) functioning, namely the updating, monitoring, and manipulation of WM representations. The EF 'shifting' is defined by processes of shifting between multiple tasks, operations, or mental sets. The EF labeled 'inhibition' in a narrow sense refers to processes of response inhibition (e.g., in a Stroop task, cf. [8]), and more broadly defined refers to general processes of interference control or executive attention [9].

Text reading and comprehension require a number of lower level cognitive processes like letter decoding and word recognition as well as higher level cognitive processes like language and discourse processes, and domain general processes such as WM and EFs [10–12]. WM and EFs are especially required for the comprehension level of text reading [11,13]. According to the influential construction-integration model by Kintsch and colleagues [14,15], text comprehension consists of an iterative sequence of two steps that each refers to a specific level of mental representation. First, in the construction step, a mental model of the propositions of the text (i.e, the textbase) is generated. This textbase that has been constructed purely text-driven is then integrated into a situation model, i.e., a mental model of what the text is about [16]. The situation model comprises information from the text as well as inferences made based on the text and prior knowledge [17]. During reading, the situation model has to be continuously updated to integrate the new information [14].

Generally, hyperlinks might affect both steps of the construction-integration cycle during reading. First, the construction step may be affected when hyperlinks interrupt the reading process and demand additional EFs like shifting and inhibition: Readers have to perform a task shift from purely reading to a decision on hyperlink selection. Additionally, inhibitory processes may be required to ignore irrelevant links and focus on relevant links. Second, when readers decide to follow a hyperlink the integration step might also be affected as the text of the following web page has to be integrated into the situation model [2,14]. Traditionally, hypertext research (for a review, see [2]), has studied the entire process of link selection and browsing through the subsequent web pages as a whole, without differentiating between load on EFs imposed by the hyperlink selection processes per se (i.e., affecting the construction step of text comprehension) and the load imposed on EFs through getting disoriented on the following web pages (i.e., affecting the integration step of text comprehension; [1,18–21]).

In the present research we were explicitly interested in load on EFs imposed by link selection processes per se, without inducing additional load due to the retrieval of subsequent hypertext pages and possible additional effects of disorientation. While the load effects of hyperlinks have been stated by several authors (e.g., [22,23]), to the best of our knowledge only one study by Fitzsimmons, Weal, and Drieghe so far has explicitly addressed the influence of links on text reading without the additional effects of load induced by displaying the following hypertext pages [24].

In this study (Experiment 2) by Fitzsimmons and colleagues [24] the authors compared reading of modified Wikipedia articles with texts that were either presented with hyperlinks (blue colored words) or without hyperlinks. Additionally the words chosen as hyperlinks were either high-frequent or low-frequent words. Participants were instructed to simply read through the texts. They had no possibility to actually select the hyperlinks (i.e., to click on the links). During reading, eye-tracking data were recorded. The authors did not find an overall effect of disruption in the reading process due to the hyperlinks. Only low frequent words marked as hyperlinks led to longer fixation durations and a rereading of previous paragraphs. This was interpreted by the authors as indication that hyperlinks may highlight important information for the reader which might be especially of relevance in case of uncommon, difficult concepts. However, as participants in this study did not have to perform selection processes (by clicking on the hyperlinks), the potential influence of actual link selection processes on reading due to a possible increase in load on EFs remained unclear.

Thus, in the current study we wanted to extend this line of hypertext reading research by focusing on the initial selection processes of words marked as hyperlinks. Specifically, we were interested in whether words marked as hyperlinks from which readers had to select appropriate ones, would induce additional load on EFs during the reading process due to the selection processes required. For this purpose we used pupil dilation and EEG alpha frequency band power as measures of load on EFs during reading in a task paradigm that allowed us to record and analyze these physiological measures in a natural reading situation. As will be outlined in the following, both measures, pupil dilation and EEG alpha frequency band power, have been shown to be sensitive for load on EFs when used in highly controlled, low-level tasks, such as working memory or attention tasks. However, only few studies have examined these load-measures in more unconstrained, natural task situations.

Examining low-level tasks it has been shown that the eye pupil dilates if EFs are required, e.g., due to demands on the EF 'updating' in an n-back task [25] or the EF 'inhibition' in a Stroop task [26]. However, the eye pupil not only dilates due to specific demands on EFs but also more generally due to load on the cognitive processing system [27,28] as well as due to increased effort [29], or even changes in emotional or motivational states [30]. Recently, a direct connection between pupil dilation and the activity of the locus coeruleus in the brain that is central to the noradrenergic system has been proposed, based on the results of fMRI outcomes [31]. This indicates a close connection between pupil dilation and general states of arousal. Thus, pupil dilation may be seen as a rather overall load measure, including aspects of effort, motivation, arousal, and emotion [30,32–36]. Depending on the environmental lighting condition, the size of the pupils varies between two to nine millimeters [32]. Changes in pupil diameter due to cognitive processing demands are rather small, normally less than one millimeter irrespective of the baseline pupil diameter. Nevertheless, this measure has been proven to be very reliable in low-level tasks [30].

Only few studies have used pupil dilation in rather complex task materials such as unconstrained, free (hypertext) reading situations [37,38]. For example, Di Stasi and colleagues [38] recorded participants’ pupil diameter while they had to perform two shopping tasks on a commercial website, either a goal-oriented search task (find and buy a specific object) or an experience-oriented search task (freely browse through the websites and possibly buy objects of own choice). In both task conditions an initial two minutes free exploration of the website without buying objects served as baseline. The authors found that in both task conditions the eye pupils significantly dilated from baseline when participants started one of the tasks. However, in addition, subjective rating scores revealed that the goal-oriented search task was experienced as being more difficult as the browsing task. This subjective difference in difficulty between tasks was not reflected in pupillary results. To conclude, these findings indicate that the sensitivity of pupil dilation as a measure of load in complex task settings is limited. Therefore, part of the research question of the present research was to examine the general sensitivity of pupil dilation as a measure of load on EFs in online reading and hyperlink selection.

EEG alpha frequency band power reflects the strength of EEG oscillatory activity and has been traditionally defined as the frequency range between eight to 13 Hz [39]. For increased demands on cognitive processing, e.g., due to load on the EF 'updating' in an n-back task [40] or due to load on the EF 'inhibition' in a Stroop task [41], oscillatory activity has been observed to desynchronize. This event-related desynchronization (ERD) due to cognitive processing load during task performance results in decreased alpha frequency band power as compared to the frequency band power of a baseline interval [42]. The alpha ERD is commonly most pronounced over parietal electrodes [43], but it can also show certain effects of lateralization depending on the task material used (e.g., a left-lateralization for linguistic task material [44]). A synchronization of oscillatory activity in the alpha frequency band range (i.e., an increase in alpha frequency band power) might either reflect the activity of a cortical idling network [45], or, as proposed more recently, the active suppression of those brain networks not required for task performance or of those that are potentially interfering with task-relevant ones [46,47]. The alpha ERD has been associated with processes of attention and semantic memory [48,49]) and generally seems to reflect cognitive processing demands when WM or EFs are demanded ([40,41]). Thus, alpha frequency band power might be particularly suited to capture cognitive processes associated with link selection as described above.

EEG measures have quite recently been started to be applied in the context of unconstraint ('real-world') text reading, mostly using eye fixation-related potentials [50–53]. We sought to extend this line of research by focusing on EEG alpha frequency band power and the use of hypertext material as task domain. Only few studies report EEG alpha frequency band power as measures of load in rather complex, free reading situations (e.g., [54,55]; see [56] for a review in the context of hypermedia research). For example, Antonenko and Niederhauser [54] used the EEG alpha frequency band power to assess the impact of leads (i.e., short previews or descriptions of succeeding websites) on cognitive load during hyperlink selection. As expected, they observed a decrease in alpha frequency band power during link selection (i.e., an alpha ERD). However, this alpha ERD was reduced for those hyperlinks that provided a preview of some initial sentences of the following hyperlinked page via mouse-over. This was interpreted by the authors to reflect reduced cognitive load during the link selection process when leads are given as compared to normal hyperlinks without leads. However, as the task conditions in this study (leads versus no-leads) also varied considerably with regard to perceptual differences, the observed effects might simply go back to perceptual confounds and not to different load situations per se (for a comprehensive overview of this critical issue in most studies using complex task materials see [57]). By carefully controlling for perceptual confounds, our study was hypothesized to tap deeper into purely load-related effects of link selection processes during text reading.

To sum up, in the present study we sought to further address and extend two currently emerging research directions: (a) a more in-depth analysis of demands on EFs in online reading and hyperlink selection, and (b) the combined recording and analysis of EEG data and pupil dilation data in a complex reading task. To the best of our knowledge a direct comparison of pupil dilation data and EEG alpha frequency band power data as measures of load on EFs has not been conducted before, neither in highly constrained (e.g., working memory) task settings, nor in rather unconstrained, free reading task settings.

As we focused on the influence of initial link selection processes during reading (i.e., during the construction step of text comprehension as described above) without hampering reading comprehension due to following web pages (i.e., the integration step of reading comprehension), we created a rather artificial hypertext reading situation that consisted of one text presented on the screen with words marked as hyperlinks but without any hyperlink functionality (i.e., no further pages could be reached). Furthermore, we simulated the link selection processes by instructing the participants to only click on context-matching words that were designed as links (see Method section for a detailed description of the task material). Although we expected our research account to tap into comparable cognitive effects of link selection during reading that might occur in genuine hypertext reading situations, one should keep in mind that we used a rather artificial hypertext-reading situation with simulated link-selection processes (see the General Discussion section for addressing the validity of our research account).

We hypothesized to observe increased load on EFs during those link selection processes (test condition) in terms of increased pupil dilation (Hypothesis 1a) and decreased alpha frequency band power (Hypothesis 1b) when comparing this test condition to a baseline condition within the same text but consisting of pure text reading. Additionally, we expected that the change of pupil dilation and the change of alpha frequency band power between baseline and test condition would be correlated. As the pupil dilation was expected to increase for increased task demands and the alpha frequency band power was expected to decrease for increased task demands (i.e., an increased alpha ERD) we expected to observe a negative correlation between the two measures (i.e., a larger increase in pupil dilation should be accompanied by larger, yet negatively signed, alpha ERD; Hypothesis 2).

In the following sections we will describe three experiments that were run to address these research questions and to carefully rule out possible alternative explanations due to confounding factors. Experiment 1 most closely simulated a hypertext reading and link selection situation. Experiment 2 and Experiment 3 ruled out possible alternative explanations of the observed results due to perceptual (i.e., word color), motor (i.e., mouse-click), or structural (i.e., sentence difficulty) confounds.

## Experiment 1

In the present research we were interested in a specific aspect of load-induction due to the availability of hyperlinks, namely the load on EFs that is induced when text reading is interrupted by words marked as hyperlinks and selection processes have to take place. In Experiment 1 we used a methodology of combined recording and analysis of eye-tracking and EEG data. This methodology allowed us to directly compare the outcomes of two physiological load-measures, pupil dilation and EEG alpha frequency band power, during natural reading of one hypertext page. Two task conditions (baseline and test condition) were implemented within the text. In Experiment 1, parts of the text requiring pure text reading served as baseline condition. Parts of the text requiring additional hyperlink-like selection processes served as test condition.

### Methods

#### Participants

Twenty-three university students (mean age = 24.83 years, *SD* = 3.20, 13 females) participated in the study and received a payment of 8 €/h. They were all native speakers of German, right-handed, and reported no neurological disorders. All participants had normal or corrected-to-normal visual acuity. The study was approved by the local ethic committee of the Knowledge Media Research Center Tuebingen. Participants gave their written informed consent at the beginning of the study. None of the participants was familiar beforehand with the task materials we used.

#### Materials and procedure

**Fig 1:**
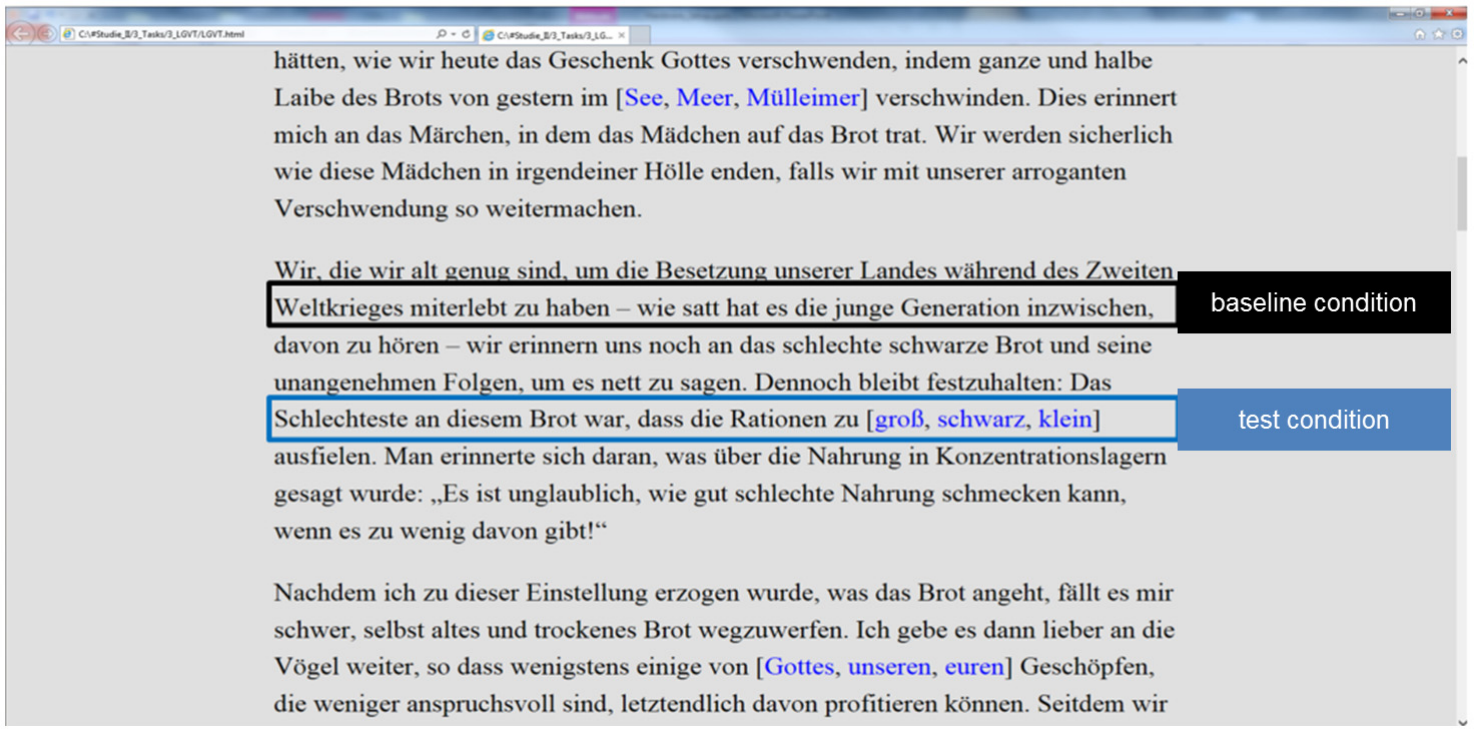
Exemplary extract of the task materials used in Experiment 1.

Task materials consisted of a text taken out of a German reading-comprehension task (the LGVT, [58]). The text was of standardized difficulty, suitable for testing reading abilities of German high-school students and thus neither over- nor undertaxing a university student sample. The text was 1727 words long. It was presented as one hypertext page in a normal web browser (Microsoft Internet Explorer). Font size was 35 points; spacing was set to 24 points. Font type was Times New Roman, font color black, and background color light gray (see Fig 1 for an exemplary part of the stimulus material used in Experiment 1).

Participants were instructed to read through the text quickly yet attentive. As in the standard LGVT task instruction, participants were informed that they would have only four minutes for text reading. We slightly deceived our participants about the actual time constraint: Although we announced a time constraint of four minutes, we let participants read through the whole text in their individual reading speed until they reached the final sentence. By announcing a hypothetical time constraint we wanted to ensure a concentrated, linear text reading process without losing data for the later analysis. A visual inspection of the eye tracking data afterwards confirmed the generally linear reading process. Only when areas of the text were reached that contained the links, participants interrupted shortly the linear reading pattern and some back and forth jumps to nearby words could be observed. A rather shallow text processing in the form of text skimming (e.g., [59]) due to the announced rather short time constraint of four minutes did not occur. This can also be inferred from the rather high reading comprehension scores obtained (see Tables 1 and 2).

**Table 1:** Pearsons' Correlations Coefficients (two-tailed) for the Physiological Measures (Difference in Alpha Power [in μV^2^/Hz] and Pupil Size [in mm] between Baseline and Test Condition) and the LGVT Reading Comprehension Score and the Total Reading Time [in minutes] of Experiment 1.

Variable	1	2	3	M	SD	N
1. Δ alpha power				-1.38	1.65	23
2. Δ pupil size	-.16			0.07	0.07	23
3. reading comprehension	.-41^+^	.16		21.17	5.62	23
4. total reading time	.16	-.11	-.80***	8.43	1.69	23

Note.

^+^ p <. 10,

* p <. 05,

*** p <. 001.

**Table 2:** Pearsons' Correlations Coefficients (two-tailed) for the Physiological Measures (Difference in Alpha Power [in μV^2^/Hz] and Pupil Size [in mm] between Baseline and Test Condition) and the LGVT Reading Comprehension Score and the Total Reading tTme [in minutes] of Experiment 2.

Variable	1	2	3	M	SD	N
1. Δ alpha power				-0.98	1.10	19
2. Δ pupil size	-.14			0.07	0.05	19
3. reading comprehension	-.50*	-.23		19.84	5.09	19
4. total reading time	.43^+^	.08	-.84***	8.52	1.90	19

Note.

^+^ p <. 10,

* p <. 05,

*** p <. 001.

At each of 23 different positions in the text three words were set in brackets. One out of the three words matched the context, the two others were distractors. For example, the exemplary text part given in Fig 1 describes the bad food supply situation of the population during World War II, and mentions a certain kind of bad black bread. The sentence in the test condition states: "The worst thing about this bread was that the food rations were too [big, black, small]". Participants had to click on the context-matching word (i.e., "small" in this case). Thus, they had to perform decision and selection processes that we hypothesized to be comparable to initial hyperlink-selection processes. The words in brackets were blue colored to simulate hyperlinks. To provide a visual feedback the blue color changed to red once a link was clicked on. There were no other effects of clicking. The 23 decision items of simulated hyperlinks were in 12 cases nouns, in 7 cases adjectives, in 2 cases verbs, and in 2 cases pronouns. The use of different word forms might resemble the distribution of actual hyperlinks for example in a Wikipedia article where also not only nouns serve as hyperlinks. Furthermore, comparable to real hyperlinks, the 23 decision items required semantic processing. None of the correct items could be inferred by syntactical or morphological processing.

The text out of a German reading comprehension task we used may be regarded as a rather artificial kind of a hypertext page. However, we favored the use of a carefully designed text of standardized difficulty like the LGVT text as task material for two reasons: Firstly, the controlled text ensured that participants' reading comprehension was challenged but not over-taxed, i.e., it ensured that the participants were cognitively neither over- nor under-loaded during reading (which otherwise might have confounded our physiological measures). Secondly, the LGVT text provided us with two behavioral measures, reading speed and reading comprehension scores, which we could use to check whether participants had attentively read the text.

The experiment started after the EEG preparation and the calibration of the eye-tracker. Written task instructions were presented as the first page on the screen. Participants reached the LGVT page via a hyperlink at the end of the task instructions. The total duration of the experiment including the technical preparation procedures was about one hour.

#### Apparatus

The experiment was run in a quiet room that was dimly lit. Participants sat in a comfortable chair in front of a 22-inch Dell monitor (1680x1050 pixels screen resolution) while their EEG and eye-tracking data were recorded. Eye-tracking data were recorded using a 250 Hz SMI (SensoMotoric Instruments) infrared remote eye-tracking system that was positioned below the monitor. A chin rest was used to avoid head movements during data recording and to guarantee a fixed distance of about 70 cm between the eyes and the eye-tracking device. The eye-tracking data were recorded at a sampling rate of 250 Hz (SMI iView X 2.7.13). The eye-tracker was calibrated using the built-in calibration routines (SMI Experiment Center, 9-point calibration) before the first page (written task instructions) appeared on the screen. EEG data were recorded from 27 electrode sites (Fp1, Fp2, F7, F3, Fz, F4, F8, FC5, FC1, FC2, FC6, T7, C3, Cz, C4, T8, CP5, CP1, CP2, CP6, P7, P3, Pz, P4, P8, O1, O2) positioned according to the international 10/20 system [60]. The right mastoid served as reference during recording. Ground electrode was positioned at AFz. Three additional electrodes were placed around the eyes for recording of the electro-occulogram (EOG). EEG data were recorded (PyCorder 1.0.2) at 500 Hz sampling rate (ActiCHamp, Brainproducts, Inc.) using active electrodes (ActiCap, Brainproducts, Inc.). Impedances were kept below 5 kOhm.

#### Data preprocessing and analysis

During preprocessing, before synchronization with the EEG data, the eye-tracking data were upsampled to 500 Hz to match the sampling rate of the EEG data. Eye-tracking data and EEG data were preprocessed and synchronized using customized Matlab scripts (Matlab 2012b, MathWorks, Inc.; EEGLAB v. 11.0.5.4b, [61], with EYE-EEG plugin, [51]). Eye blink artifacts (missing data points) in the eye-tracking data were corrected using linear interpolation. The continuous EEG data were filtered (low-pass 40 Hz, high-pass 0.5 Hz, linear finite impulse response filters). EOG artifacts were corrected using independent component analysis (ICA) decompositions. Independent components (ICs) identified as EOG-ICs by visual inspection were rejected. EEG data were re-referenced to average reference.

The combined continuous EEG and eye-tracking data were split in epochs of two seconds length for the two task conditions of interest, baseline and test condition. Only first visits of these epochs were considered for analyses. However, a visual inspection of the eye-tracking data showed a rather linear text reading process with no severe view jumps (e.g., regressions to previously read sentence lines). For the baseline condition (pure text reading) the epochs were defined through areas of interest (AOIs) positioned around text lines that lay maximally in between parts of the text where link selection took place. On average 2.9 text lines (range: 1 to 7) were between the test and the baseline conditions. Post-hoc visual inspection of the eye-tracking data ensured that even in cases where the two conditions were separated by only one line, the two conditions were not confounded by each other. For the test condition (link selection) the epochs were defined as ending 500 ms before the mouse click to avoid motor artifacts in the EEG data [62] and to minimize the motor differences between baseline and test condition. In total, 46 data epochs were created, 23 epochs for each task condition. An automatic artifact removal was performed with respect to the EEG data: Epochs that exceeded ±100 μV were excluded from further analyses [63]. By using this criterion, epochs containing severe artifacts (e.g., muscle artifacts) were excluded. No further artifact removal or correction was performed on the EEG data.

For the eye-tracking data, the mean pupil size was calculated by averaging the left and right pupil data. These mean pupil data were further averaged for each of the 2 s epochs and then averaged over all epochs for each task condition. For the EEG data, EEG frequency band power was calculated using fast-fourier transforms (FFTs) for the entire epoch lengths in the alpha frequency band spectrum (8 Hz to 13 Hz). The alpha frequency band power was then averaged individually over all epochs for each task condition.

### Results

#### Behavioral data

Reading comprehension scores were calculated according to the LGVT manual: For the initial four minutes of text reading each correctly selected word counted as +2 points, each wrongly selected word as -1 point. Points were summed up for each participant. Total reading time was defined as the entire time participants read through the LGVT page until they reached the final sentence. Reading comprehension score and total reading time are given in Table 1. These measures show that participants attentively read the text, yet not being cognitively overloaded (i.e., participants' reading comprehension scores are in the upper third of the common LGVT outcomes, a result quite typical for university students).

#### Physiological data

**Fig 2:**
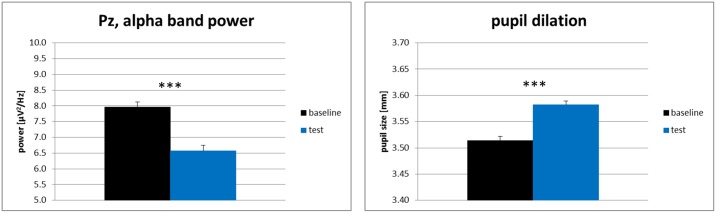
Mean alpha (8–13 Hz) frequency band power at electrode Pz and mean pupil dilation of Experiment 1. Note. *** indicate p <. 001, black error bars indicate +1 standard error of the mean.

**Fig 3:**
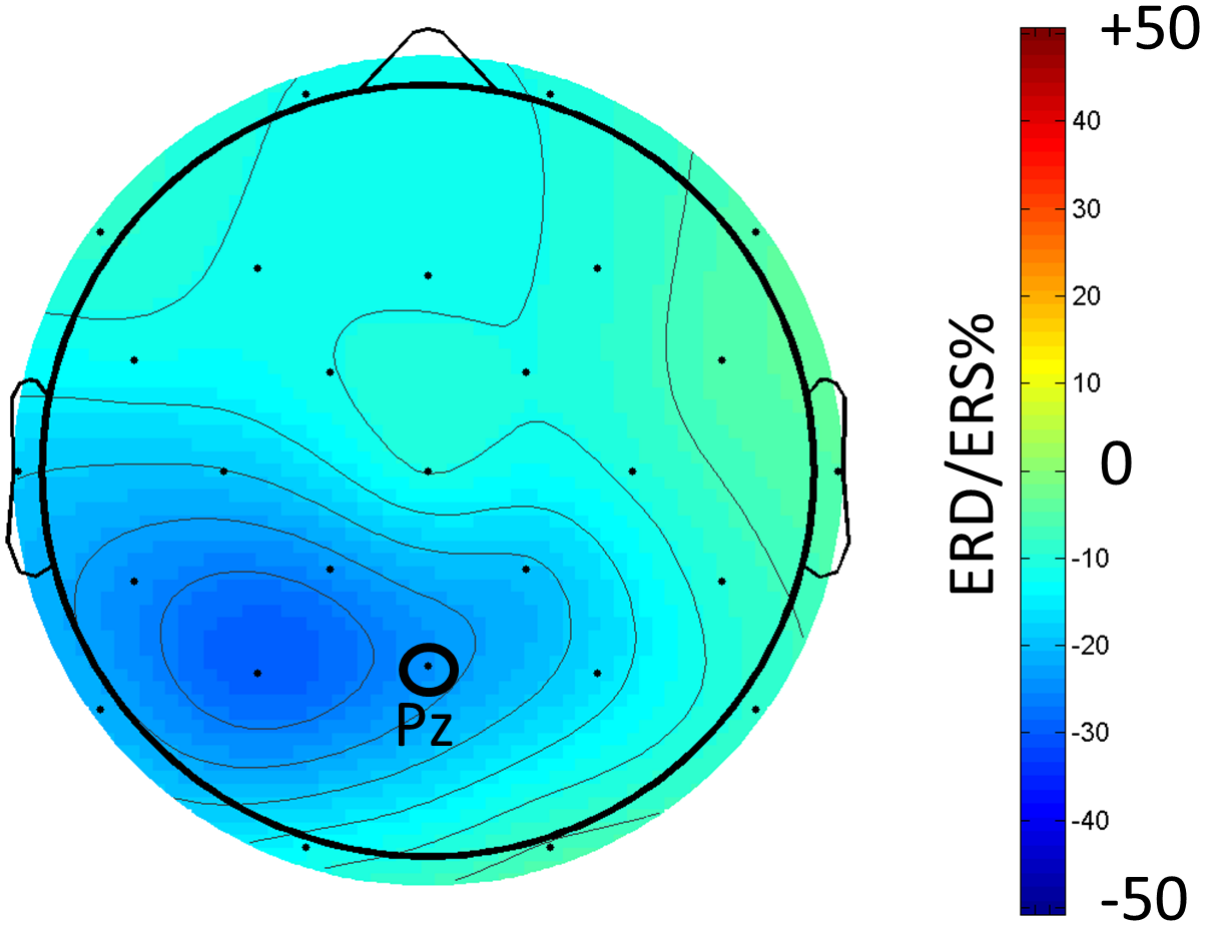
Topoplot of the percentual changes of alpha (8–13 Hz) frequency band power between baseline condition and test condition for Experiment 1. Percentual frequency band power changes (i.e., the event-related desynchronization/synchronization, ERD/ERS%) were calculated after the formula given in [42].

For each physiological measure (EEG alpha frequency band power at electrode Pz and pupil size), we computed separate one-factorial repeated measures ANOVAs (baseline condition vs. test condition). The results for the physiological variables are shown in Fig 2. We restricted this analysis to the parietal electrode Pz as alpha frequency band power effects are generally reported to be most pronounced at parietal electrodes with Pz as representative (e.g., [64,65]). To further explore topographical differences of the alpha frequency band power effects as indicated by the topoplot in Fig 3, an additional 3-way repeated-measures ANOVA was conducted with the factors hemisphere (left / right), electrode site (frontal / parietal) and task condition (baseline / test). For post-hoc pairwise comparisons all *p*-values were Bonferroni corrected for multiple comparisons. Level of significance was set at α = .05 for all analyses and partial eta-square (*η*_p_^2^) is reported as a measure of effect size.

For the pupil dilation data we found a main effect of task condition, *F*(1, 22) = 21.85, *p* <. 001, *η*_p_^2^ = .50. In line with hypothesis 1a, pupil sizes in the test condition (*M* = 3.58 mm, *SD* = 0.32) were significantly larger as compared to pupil sizes in the baseline condition (*M* = 3.51 mm, *SD* = 0.32). We also found a main effect of task condition for the alpha frequency band power at electrode Pz, *F*(1, 22) = 16.05, *p* = .001, *η*_*p*_^*2*^ = .42. As expected by Hypothesis 1b, the alpha frequency band power in the test condition (*M* = 6.58 μV^2^/Hz, *SD* = 2.16) was significantly lower as in the baseline condition (*M* = 7.96 μV^2^/Hz, *SD* = 2.84).

The 3-way repeated-measures ANOVA strengthened these results (cf. the topoplot in Fig 3 showing the percent change in alpha frequency band power between baseline and test condition for all electrodes plotted over the scalp). We observed a main effect of hemisphere, *F*(1, 22) = 11.32, *p* = .003, *η*_*p*_^*2*^ = .34, with electrodes over the left hemisphere generally showing lower alpha frequency band power values (left: 7.94 μV^2^/Hz vs. right: 8.40 μV^2^/Hz), and a main effect of task condition, *F*(1, 22) = 10.13, *p* = .004, *η*_*p*_^*2*^ = .32, with the test condition showing significantly lower alpha frequency band power values as compared to the baseline condition (test: 7.82 μV^2^/Hz vs. baseline: 8.40 μV^2^/Hz).

However, these main effects were qualified by a significant interaction between task condition and hemisphere, *F*(1, 22) = 20.61, *p* <. 001, *η*_*p*_^*2*^ = .48. This interaction was due to the fact that the alpha frequency band power decreased more strongly in the left than in the right hemisphere between baseline and test condition (left:-.88 μV^2^/Hz, *p* = .001, right:-.52 μV^2^/Hz, *p* = .028) and particularly due to the fact that only in the test condition alpha frequency band power between left and right hemisphere differed (test condition:-.64 μV^2^/Hz, *p* <. 001, baseline condition:-.29 μV^2^/Hz, *p* = .058). These findings are in line with other studies using linguistic task material that observed left lateralized effects, as will be discussed in the general discussion section of this paper.

Additionally we observed a significant interaction between task condition and electrode site, *F*(1, 22) = 15.41, *p* = .001, *η*_*p*_^*2*^ = .41. This interaction resulted from a larger decrease of alpha frequency band power due to test condition at parietal-occipital electrode sites as compared to baseline condition (parietal-occipital:-.91 μV^2^/Hz, *p* <. 001, frontal:-.50 μV^2^/Hz, *p* = .047). These findings are in line with typically observed largest effect sizes of alpha frequency band power changes at parietal-occipital electrodes (e.g., [43]).

#### Correlational data

As we were further interested in whether changes in pupil dilation and changes in EEG alpha frequency band power were measures of comparable sensitivity for changes in load on EFs due to the selection processes in the test condition as compared to the baseline condition, we calculated the mean differences between test and baseline condition (i.e., test condition subtracted from baseline condition) for the mean pupil sizes and the mean alpha frequency band power at electrode Pz (cf., Table 1). We calculated Pearson's correlation coefficients (two-tailed) for these two physiological measures (difference values), as well as the two behavioral measures, i.e. the LGVT reading comprehension score and the total reading time. Results of the correlational analyses are given in Table 1. Contrary to Hypothesis 2, we observed no significant correlation between the amount of change in alpha frequency band power and pupil dilation. Interestingly, however, there was a marginally significant negative correlation between participants’ LGVT reading comprehension score and the difference value of alpha frequency band power between baseline and test condition. Participants with a higher LGVT reading comprehension score showed a more pronounced decrease in alpha frequency band power (i.e., more negative difference values). Finally, in accordance with the typical outcomes of the LGVT, we additionally found a strong negative correlation between the LGVT reading comprehension scores and the total reading times of the text. Participants who showed higher reading comprehension scores were also faster in reading.

### Discussion

To sum up, in Experiment 1 both physiological measures showed the expected outcomes: When comparing parts of the text that required purely reading (baseline condition) with parts of the text where participants had to interrupt reading and to perform hyperlink-like selection processes (test condition), we observed a significant increase in pupil size as well as a significant decrease of alpha frequency band power. These results are in accordance with our above stated Hypotheses 1a and 1b that the hyperlink-like selection processes in the test condition imposed additional load on EFs as compared to the pure reading situation in the baseline condition.

However, there are two possible alternative explanations that may account for the observed results. As will be outlined in the following the results might go back to motor or perceptual differences between baseline and test condition and not to cognitive processes per se. First, participants' motor activity differed between baseline an test condition. In the baseline condition, participants were reading only whereas in the test condition they had to perform a mouse-click on a word. Although we used a data epoch for data analysis that ended 500 ms before the mouse-click, we cannot fully rule out the possibility that differences in motor activity (or motor preparation processes) have confounded our results and thus serve as an alternative explanation. Second, in the baseline condition all words were black colored whereas in the test condition the words that could be selected were blue-colored and changed their color to red after participants clicked on them. These perceptual differences could also be responsible for the changes in pupil dilation. To rule out these possible effects of the perceptual and motor confounds in Experiment 1, we conducted a second experiment with slightly modified task materials.

Moreover, contrary to Hypothesis 2, we observed no significant correlation between the amount of change in alpha frequency band power and pupil dilation. This was unexpected as separate ANOVAs of pupil dilation data and EEG alpha frequency band power data both showed the expected outcomes, when comparing the two task conditions (baseline and test condition). Therefore we would have expected to find a clear negative correlation between the difference values of the two physiological measures, as the ANOVAs had shown that the pupil size increased significantly from baseline to test condition whereas the alpha frequency band power decreased significantly from baseline to test condition. One potential reason for this unexpected outcome might be the potential perceptual and motor confounds mentioned above, which might have affected the physiological variables differently. For example, one could hypothesize that the different color of the words in the test condition could affect pupil sizes but not alpha frequency band power, thus masking the expected correlation between these two measures.

Finally, we found a trend for a negative correlation between the LGVT reading comprehension score and the difference value of alpha frequency band power between baseline and test condition. Participants with a higher LGVT reading comprehension score showed a more pronounced decrease in alpha frequency band power (i.e., more negative difference values). This might underline the character of alpha frequency band power as a valid measure of essential cognitive processes. If participants were cognitively more engaged, they showed a higher reading comprehension score. In contrast, no such correlation was shown for pupil dilation.

## Experiment 2

In Experiment 2 we modified the baseline and test condition of Experiment 1 to avoid possible perceptual-motor differences between them that might have confounded our results in Experiment 1. Thus, in Experiment 2 participants had to perform a mouse-click on a word in the baseline condition in a comparable manner as in the test condition. Furthermore, we changed the color of the words in the test condition to black and removed the color-change to red of words that have been clicked on.

### Methods

#### Participants

Twenty additional university students (mean age = 24.90, *SD* = 3.23, 9 females) participated in Experiment 2. The general subject pool was the same as in Experiment 1 and the same constraints, incentives, and formal procedures were applied. None of the participants had attended Experiment 1 or was familiar with the LGVT task. One participant had to be excluded from data analysis due to technical problems during data acquisition and partly missing data.

#### Materials and procedure

**Fig 4:**
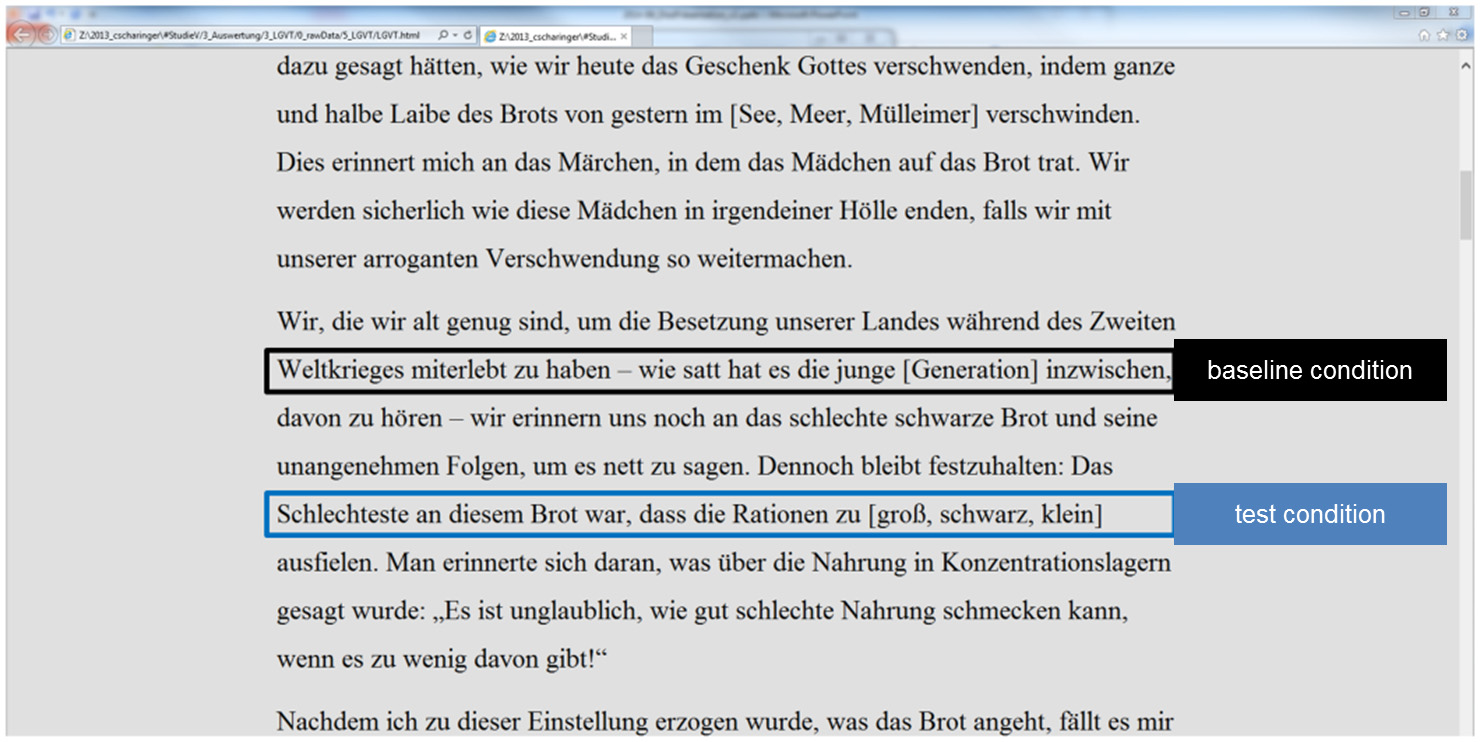
Exemplary extract of the task materials used in Experiment 2.

Task material and presentation was the same as described for Experiment 1 with the following modifications aiming at reducing any motor or perceptual differences between baseline condition and test condition as far as possible: We modified the baseline condition insofar that participants had to click on a single word set in brackets in the baseline condition (instead of purely text reading as in Experiment 1). Furthermore, to avoid any perceptual differences between baseline condition and test condition, the three words in brackets in the test condition were perceptually equalized to the entire text (i.e., black color, no color change when mouse-click was performed). In doing so, we assured in Experiment 2 that any observed difference between test condition and baseline condition should only be due to additional selection processes that were hypothesized to load more on EFs in the test condition as compared to the baseline condition. See Fig 4 for an exemplary part of the task material. As an additional minor modification, we slightly increased the spacing between sentence lines to 28.8 points. This allowed us to more easily define areas of interest that included single sentence lines.

#### Data preprocessing and analysis

Data preprocessing and analysis steps were identical to Experiment 1 with the only difference that the epoch alignment in the baseline condition in Experiment 2 was also related to the (now available) mouse-click. This equals the epoch alignment of the test condition (i.e., the 2 s epochs used for data averaging and analysis ended 500 ms before the mouse-click in both task conditions) and minimizes possible non-cognitive differences between baseline and test condition.

### Results

#### Behavioral data

The average LGVT reading comprehension score and the average total reading time are given in Table 2. These behavioral variables were comparable to Experiment 1, again indicating an attentive yet non-overloaded reading process of the participants.

#### Physiological data

**Fig 5:**
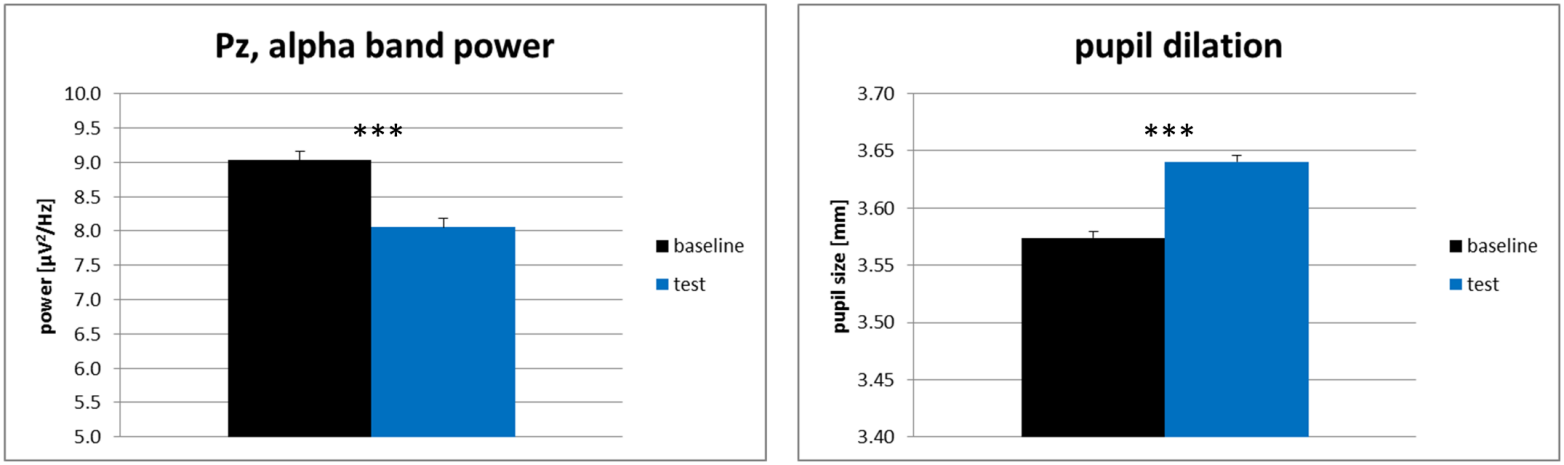
Mean alpha (8–13 Hz) frequency band power at electrode Pz and mean pupil dilation of Experiment 2. Note. *** indicate p <. 001, black error bars indicate +1 standard error of the mean.

As in Experiment 1, we found a main effect of task condition for the pupil dilation data, *F*(1, 18) = 32.61, *p* <. 001, *η*_*p*_^*2*^ = .64. Pupil sizes in the test condition (*M* = 3.64 mm, *SD* = 0.36) were significantly larger as compared to pupil sizes in the baseline condition (*M* = 3.57 mm, *SD* = 0.34), confirming Hypothesis 1a. We also found a main effect of task condition for the alpha frequency band power, *F*(1, 18) = 15.00, *p* = .001, *η*_*p*_^*2*^ = .45. As expected by Hypothesis 1b, the alpha frequency band power in the test condition (*M* = 8.05 μV^2^/Hz, *SD* = 2.03) was significantly lower as in the baseline condition (*M* = 8.87 μV^2^/Hz, *SD* = 9.03). The outcomes of these measures can be seen in Fig 5.

**Fig 6:**
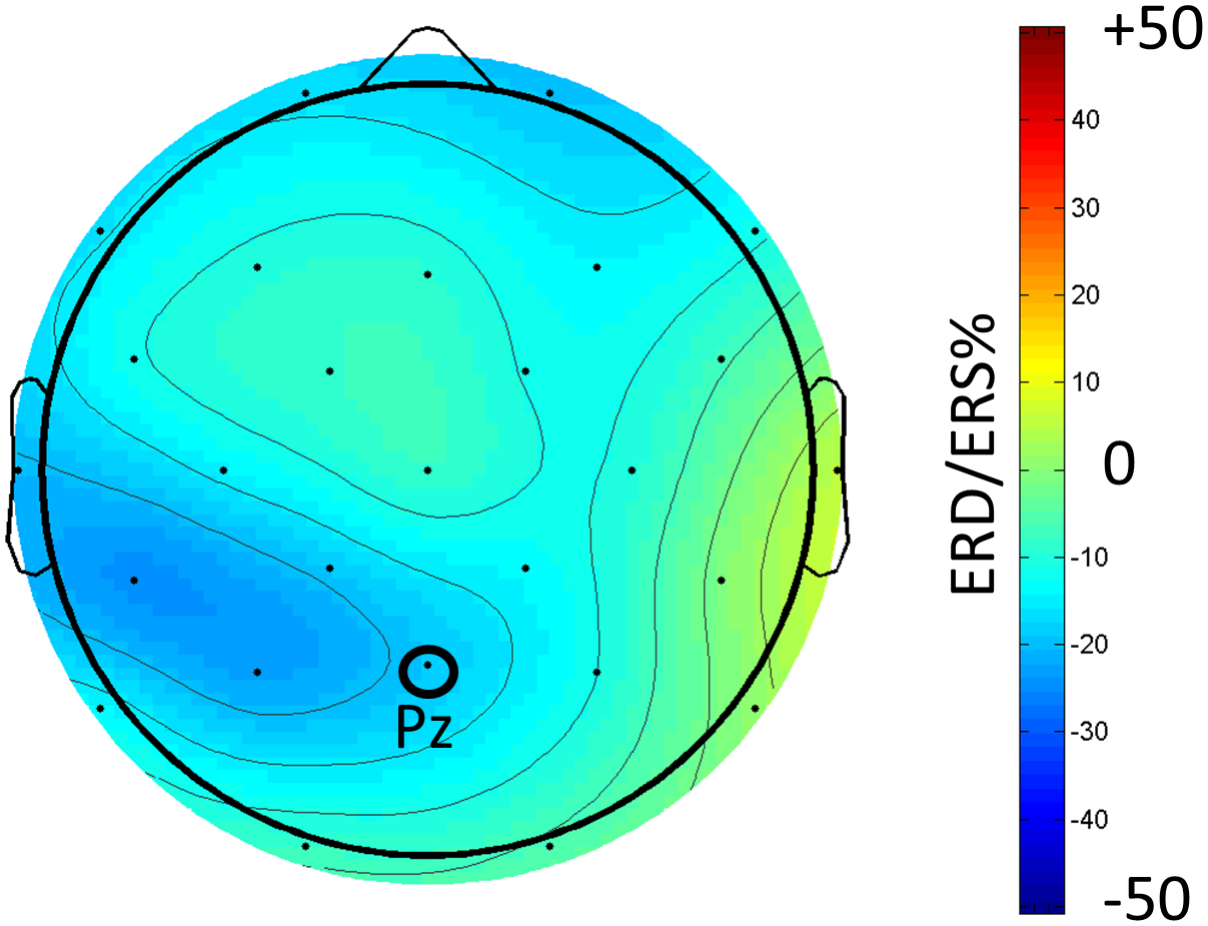
Topoplot of the percentual changes of alpha (8–13 Hz) frequency band power between baseline condition and test condition for Experiment 2. Percentual frequency band power changes (i.e., the event-related desynchronization/synchronization, ERD/ERS%) were calculated after the formula given in [42].

The 3-way repeated-measures ANOVA revealed a main effect of hemisphere, *F*(1, 18) = 8.55, *p* = .009, *η*_*p*_^*2*^ = .32, and a main effect of electrode site, *F*(1, 18) = 95.68, *p* <. 001, *η*_*p*_^*2*^ = .84. However there was also a significant interaction between hemisphere and electrode site, *F*(1, 18) = 20.99, *p* <. 001, η_p_^2^ = .54. This interaction was due to the fact that only for parietal-occipital electrode sites alpha frequency band power was in general significantly smaller in the left hemisphere than in the right hemisphere (frontal-left: 7.08 μV^2^/Hz vs. frontal-right: 6.89 μV^2^/Hz, *p* = .09; parietal-left: 8.42 μV^2^/Hz vs. parietal-right: 9.23 μV^2^/Hz, *p* <. 001). This hemispheric difference did not occur at frontal electrode sites. The significant main effect of electrode site and the interaction between hemisphere and electrode site were not present in Experiment 1. We do not have a concise explanation of this difference between Experiment 1 and Experiment 2. However, and more importantly, comparable to Experiment 1 we found an interaction between task condition and hemisphere, *F*(1, 18) = 8.60, *p* = .009, *η*_*p*_^*2*^ = .32. Although all post-hoc pairwise comparisons were significant (*p* <. 001), numerically, alpha frequency band changes for test condition in comparison with baseline condition were larger in the left than in the right hemisphere (left:-.82 μV^2^/Hz, *p* <. 001, right:-.65 μV^2^/Hz, *p* = .001), which mirrors the results of Experiment 1. Additionally hemispheric differences were larger in the test condition as compared to the baseline condition (test:-.40 μV^2^/Hz, *p* = .006, baseline:-.22 μV^2^/Hz, *p* = .024). This again indicates, in line with the literature, that the strongest alpha frequency band effects occur at parietal-occipital electrodes (e.g., Gevins et al., 1997) and that left-lateralized effects may be typical for linguistic task material. Fig 6 visualizes the topographic distribution of the alpha frequency band change between baseline and test condition on the scalp.

#### Correlational data

In line with Experiment 1 we found no significant correlations between the difference values of both physiological measures (i.e., the difference value of mean pupil size between baseline and test condition and the difference value of mean alpha frequency band power between baseline and test condition, see Table 2). Also in line with the results of Experiment 1, we found a significant negative correlation between the LGVT reading comprehension score and the difference value of the alpha frequency band power between baseline and test condition. Furthermore, there was a trend for a positive correlation between total reading time and the alpha power difference value. Finally, the observed negative correlation between LGVT reading comprehension scores and total reading times also resemble the results of Experiment 1 (cf. Tables 1 and 2).

### Discussion

To sum up, the outcomes of the physiological measures of Experiment 2 were comparable to the results of Experiment 1. The results strongly support the hypothesis that selection processes in the test condition led to additional load on EFs. This increased load was measurable by increased pupil dilation data (Hypothesis 1a) as well as decreased EEG alpha frequency band power (Hypothesis 1b). As we carefully controlled the task material in Experiment 2 for any motor or perceptual confounds, we can rule out the alternative explanation of our results raised in Experiment 1 based on possible perceptual-motor confounds. However, apart from perceptual-motor confounds a third alternative explanation might be formulated: The parts of the text included in the test condition might per se have happened to be more difficult as compared to the parts of the text included in the baseline condition. To also rule out this alternative explanation, we conducted Experiment 3.

Furthermore, in line with Experiment 1 we found no significant correlations between the difference values of the two physiological measures (i.e., the difference value of mean pupil size between baseline and test condition and the difference value of mean alpha frequency band power between baseline and test condition). As we carefully controlled baseline and test condition for perceptual-motor confounds in Experiment 2, we may rule out any explanation of this non-correlation due to perceptual-motor influences that affected one measure but not the other. We will discuss this unexpected outcome in the general discussion section of this paper.

Finally, also in line with the results of Experiment 1, where we observed a trend for a negative correlation between the LGVT reading comprehension score and the difference value of the alpha frequency band power between baseline and test condition, in Experiment 2 we found a significant negative correlation between these two variables. In Experiment 2 this negative correlation was furthermore accompanied by a trend for a positive correlation between total reading time and the alpha power difference value. As hypothesized above, these results may be interpreted to underline the character of alpha frequency band power as reflecting essential cognitive processes. The more successfully participants performed the task (as indicated by higher LGVT reading comprehension scores and lower total reading times) the more pronounced was the difference of the oscillatory alpha frequency band activity between baseline and test condition (i.e., the more negative were the difference values between baseline and test condition).

## Experiment 3

**Fig 7:**
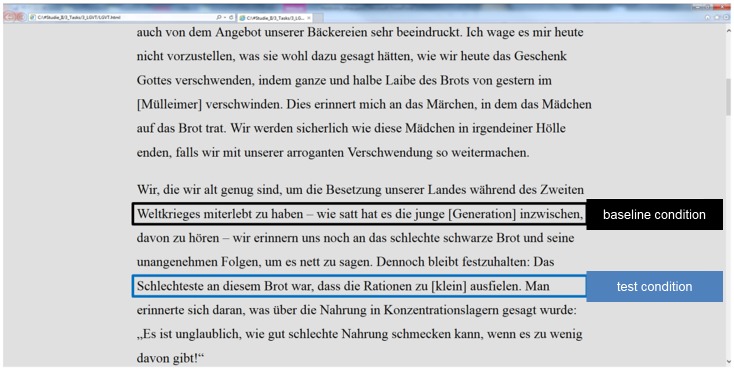
Exemplary extract of the task materials used in Experiment 3.

Experiment 3 was conducted to exclude potential differences between baseline and test condition due to differences in difficulty of the text parts used in these two conditions. Therefore, we modified the test condition of Experiment 2 to resemble the task of the baseline condition (i.e., we eliminated the word selection processes): In the test condition (as well as in the baseline condition) participants had to click on one single word in brackets (cf. Fig 7 for an exemplary part of the task material used in Experiment 3). We expected the textual difficulty in both conditions to be equal, i.e. we expected to observe no differences between baseline and test condition in Experiment 3. As pupil dilation data and EEG alpha frequency band power data had been showing similar load-related effects, for reasons of time-efficiency we recorded and analyzed only pupil dilation data in Experiment 3.

### Methods

#### Participants

Twenty-four additional university students (mean age = 23.38, *SD* = 2.55, 16 females) participated in Experiment 3. The general subject pool was the same as in Experiment 1 and Experiment 2 and the same constraints, incentives, and formal procedures were applied. None of the participants had attended Experiment 1 or Experiment 2 or was familiar with the LGVT task.

#### Materials and procedure

Task material and presentation was the same as described for Experiment 2 with the following modification: Instead of presenting three words in brackets in the test condition only the one context-matching word was presented. Thus the task in the test condition was identical to the task in the baseline condition (see Fig 7 for an exemplary extract of the task materials used). In both conditions participants had to simply click with the mouse cursor on the word in brackets. The baseline condition was identical to Experiment 2.

#### Data preprocessing and analysis

Data preprocessing and data analysis steps were identical to Experiment 2 for the eye-tracking data. Data epochs of 2 seconds length ending 500 ms before the mouse-clicks in both task conditions were used for data analysis.

### Results and Discussion

#### Behavioral data

Participants had a total mean reading time of about 7.24 minutes (*SD* = 1.67) which was slightly shorter than in Experiment 1 and Experiment 2 (cf. Tables 1 and 2). This result is not surprising due to the fact that participants' task in Experiment 3 was less complex as in the two previous experiments. Still, the overall reading time was not severely different from the two other experiments indicating that the participants were thoroughly reading the text, despite the 'easy' task.

#### Physiological data

**Fig 8:**
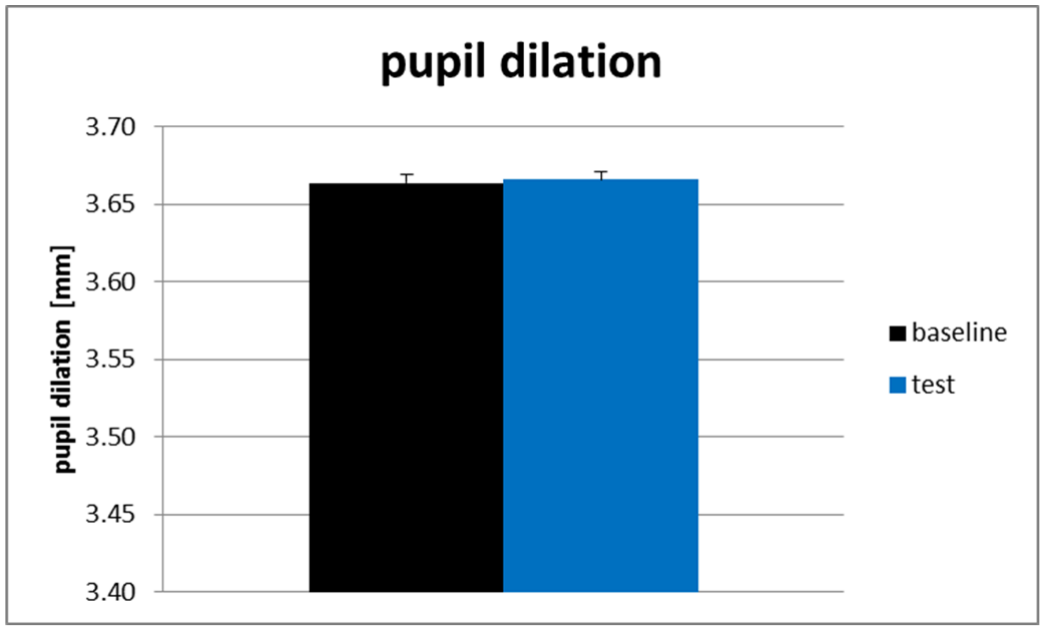
Mean pupil dilation of Experiment 3. Note. black error bars indicate +1 standard error of the mean.

We conducted a one-factorial repeated-measure ANOVA for the pupil size data of the two task conditions (baseline versus test condition). As expected we found no significant difference between pupil sizes in the baseline condition (*M* = 3.66 mm, *SD* = 0.35) and the test condition (*M* = 3.67 mm, *SD* = 0.36), *F*(1, 23) = .04, *p* = .85. In line with our expectations, this indicates that the text was of comparable difficulty in the baseline and in the test condition. The results for the pupil dilation are shown in Fig 8. To conclude, these results can rule out that the observed findings of Experiment 1 and Experiment 2 were based on potentially confounding perceptual (i.e., word color), motor (i.e., mouse-click) or structural (i.e., sentence difficulty) differences between baseline and test condition.

## General Discussion

In the present research we aimed at examining increased load on EFs during hyperlink-like selection processes in online text reading. In particular, we were interested in effects of initial link-selection processes without inducing additional load due to subsequent hypertext pages. We used a methodology of combined EEG and eye-tracking data recording and analysis that allowed us to compare two physiological load-measures, namely pupil dilation and EEG alpha frequency band power in the online hypertext-like reading situation.

As expected, both physiological measures were sensitive to increased load on EFs during hyperlink-like selection processes, confirming Hypotheses 1a and 1b. When comparing baseline and test condition, both in Epxeriment 1 and in Experiment 2 we found significantly increased pupil dilation as well as decreased alpha frequency band power for the test condition. These results are in line with studies that manipulated load on EFs in highly controlled working memory or attention tasks and that found increased pupil dilation and decreased alpha frequency band power for increased load conditions (cf., [26,43]). Thus, our study indicates that both measures, pupil dilation and EEG alpha frequency band power, seem to be sufficiently sensitive to also detect changes in load on EFs in an online reading situation.

Experiment 1 most closely simulated a hypertext-reading and hyperlink-selection situation. Participants read a text (baseline condition) and at different parts of the text they had to perform hyperlink-like selection processes (test condition). Experiment 2 and Experiment 3 ruled out possible alternative explanations of the observed results that were based on potentially confounding perceptual (i.e., word color), motor (i.e., mouse-click), or structural (i.e., sentence difficulty) differences between baseline and test condition. Thus, we can conclude that the observed differences we consistently found between baseline and test condition in Experiment 1 and Experiment 2 indeed reflect changes in the load-situation that may be related to increased demands on EFs. These findings extend traditional hypertext research that in general does not distinguish between the load imposed by hyperlink-selection processes per se (as examined in our study) and the load imposed by the additional information of subsequent web pages or the potentially experienced disorientation due to non-linear navigation processes between different pages in a hypertext [2]. Thus, our results can be seen as an empirical validation of the often implicitly made assumption in hypertext research that the presence of links per se might increase the load-situation and therefore might hamper text reading and comprehension (e.g., see [2]).

As the links we used did not lead to any subsequent text page, in our study mainly the construction step of text comprehension might have been affected (i.e., the creation of a textbase). The updating of a situation model of a text (i.e., the integration step of reading comprehension) in contrast might especially be influenced by additional information of subsequent web pages [2,14]. However, although this reasoning might sound plausible, we did not test for effects on reading comprehension by using text comprehension questions afterwards that might have allowed disentangling text comprehension with respect to propositions or inferences (i.e., textbase or situation model construction). Clearly, in future studies the influence of link-selection processes on reading comprehension should be assessed as well.

Some further limitations of the present research have to be addressed. First, as described in the introduction, conceptually it may be justified to assume that link selection during text reading would increase load on EFs. The EF shifting may be loaded because of the task-set shift from purely reading to selection and decision processes when links are encountered. In addition, the EF inhibition may be required when irrelevant links have to be ignored. However, we did not specifically manipulate load on EFs during link selection. In future research such specific manipulation of EFs during link selection might, for example, be done by presenting links of different relevance for a certain information gathering goal. Depending on the relevance of the links, the EF inhibition might be differently loaded, as for example clearly irrelevant links might be easier to ignore than links that are of mixed relevance (see [1] as an exemplary study that manipulated the relevance of links). The research methodology of combined EEG and eye-tracking data analysis might be valuably used in future studies implementing such a fine-grained manipulation of EFs. Yet, our research can serve as an initial step to advance hypertext research by showing increased cognitive demands during initial link-selection processes that might conceptually be linked to load on EFs and that can be assessed by the physiological measures pupil dilation and EEG alpha frequency band power.

Another limitation of the current research to be addressed is the ecological validity of our research paradigm that might be questioned. This is first because we used non-functional links (i.e., links that did not lead to a subsequent web page), second because we presented each time three links in direct sequence, which might be rather uncommon for hypertext reading (but which may be the typical situation readers are confronted with in case of web-search results), and third because of the for hypertexts rather unnatural linear reading situation. With respect to the first two critical aspects, we are confident that the word-selection in our paradigm in principal leads to load-related effects that are comparable to those effects that are essential in genuine hyperlink selection, such as an increased load on EFs due to performing task shifts from purely reading to decision-making, the inhibition of irrelevant words, and the selection of relevant ones. A logical next step would be to use the methodology of a combined EEG and eye-tracking data recording and analysis in a more realistic hypertext reading and browsing situation. As mentioned above, this methodology might also be used to differentiate on a fine-grained level between load imposed by different kinds of hyperlinks, e.g. task-relevant versus task-irrelevant (but nevertheless interesting) hyperlinks [1,66]. Clearly, such follow-up studies that explicitly manipulate load on different EFs during link selection will be needed to directly test our assumption that link-selection raises demands on EFs.

With respect to the third critical aspect, the task paradigm we used afforded a rather linear text reading (which was confirmed by our visual inspection of participants' eye-tracking data) that might be different from genuine online hypertext reading. Depending on the hypertext-material used, different reading patterns have been observed for online text reading. One classical example are the F-shaped reading patterns that Nielsen and colleagues observed in eye-tracking studies of real hypertext pages (e.g., [67]). Duggan and colleagues [59,68] observed reading patterns of text skimming (i.e., selectively scanning of text parts) when the time to read an online text was limited. Such time-pressure may be the typical situation in online web reading, when a huge amount of web pages addressing a certain topic are available and have to be skimmed for relevance. Reader and Payne ([69], see also [59,68]) observed an online text reading strategy termed satisficing: Parts of the text (or different texts) are skimmed through until the relevant parts of the text (or texts) are reached. These are then read more thoroughly until the individual information gain is reached. Although in the current research we announced a time-limit for text reading, the task instruction afforded our participants to apply a linear text reading strategy. Therefore, one has to keep in mind that other text reading strategies might occur in online text reading when no such task instruction is given. This has to be taken into account in future studies that should use more realistic hypertext materials that additionally might provide more realistic hypertext reading situations with respect to reading strategies than the present research. Nevertheless, given that an online text adequately meets the readers' information demands, a linear text reading like the one in our current research still might occur [69].

Despite these limitations, the current research showed the general sensitivity of pupil dilation and alpha frequency band power for an increased load-situation during link-selection. Surprisingly, however, the change in pupil dilation and the alpha frequency band power change did not correlate (Experiment 1 and Experiment 2). This outcome was rather unexpected given that the general load effect was observed for both measures. More precisely, as pupil dilation increased and alpha frequency band power decreased for increased load, we expected to observe a negative correlation between the two measures (cf., Hypotheses 2). At least two possible explanations for the observed non-correlation might be hypothesized.

First, the non-significant correlation between the two measures may indicate that they were sensitive to different aspects of load induction during hyperlink-like selection processes. Yet, we may only speculate which different aspects of load induction this may be. Pupil dilation may function as a more global load measure that also includes motivational or emotional aspects of load (e.g., [30,34–36]). This interpretation is corroborated by the non-existent correlation between reading comprehension scores and pupil dilation. In contrast, we observed a negative correlation between LGVT reading comprehension score and EEG alpha frequency band power change. This strengthens the assumption of alpha oscillatory activity being a cognitive correlate: For participants that showed higher reading comprehension scores, we observed a stronger decrease in alpha oscillatory activity. This is in line with current literature reporting a stronger alpha ERD (i.e., decrease in alpha band power) associated with higher semantic memory performance [49]. Generally, as discussed in the introduction, alpha ERD has been related to purely cognitive processes like working memory functioning, attention, and inhibitory control [46,48,49,70,71]. The interpretation of alpha frequency band power as a more sensitive measure of cognitive processes than pupil dilation may be corroborated further by the observation that in both experiments (Experiment 1 and 2) of the present research the alpha frequency band power effect was topographically maximal over left-hemispheric, parietal electrodes. This is in line with studies reporting left-lateralized alpha frequency band power effects for linguistic task material [44,72]. Noteworthy, however, based on our current results, we cannot infer that alpha frequency band power and pupil dilation might be sensitive for different EFs. Rather, the two measures might be sensitive for different aspects of load on EFs: Alpha frequency band power might be sensitive to purely cognitive aspects of load on EFs whereas pupil dilation might additionally be sensitive for emotional or motivational aspects of load on EFs [30,34,35].

Individual differences may be a second explanation for the non-existing correlation observed between the two physiological measures. In some participants pupil dilation might be the 'better' measure to detect a changed load situation, whereas in other participants EEG alpha frequency band power might be the 'better' one. If this result generally turns out to be true, it has important consequences for the entire research area of 'neuro-ergonomics' [73–75], where researchers try to detect participants' load situations using physiological measures for evaluating and adapting human-computer interfaces. For this research area our results may suggest to collect several different physiological measures and to select the most sensitive one individually later on. However, as mentioned above, these interpretations and conclusions are somewhat speculative at this point. Clearly, further research should be conducted with combined EEG and eye-tracking data recording in order to directly compare pupil dilation data and EEG frequency band data in diverse task settings and to study more closely their interlinked yet different nature. Although a few other studies have also recorded EEG data and pupil dilation data simultaneously using rather complex task materials, to the best of our knowledge correlations between EEG frequency band measures and pupil dilation measures have never been calculated or reported ([25,76–78]; but see some basic research studies mainly focusing on EEG event-related potentials for comparison with pupil dilation, e.g., [79–82], as referred to below).

To conclude, the present research may serve as an initial step with respect to two currently emerging research directions: (a) a more in-depth analysis of load on EFs in hypertext reading and hyperlink selection, and (b) the combined recording and analysis of EEG frequency band data and pupil dilation (even in 'real-world' tasks) and the exploration of different aspects of load they capture. With respect to (a), we again have to underline that we are well aware of the artificial hypertext situation in the current research as discussed above that may call for conducting additional studies. With respect to (b), we may also suggest further studies of combined EEG frequency band power analysis and pupil dilation. For this research direction using more controlled, low-level tasks may prove to be valuable. Indeed, there are some initial studies of combined EEG data and pupil dilation data analysis in basic research, reporting correlations between pupil diameter and certain EEG event-related potentials, like P300 and N400 [80–82] or between stimulus-evoked pupil dilation and EEG alpha activity [79]. These studies often use single-subject stepwise correlational analysis over the time course of trials, which were beyond the scope of the present paper. However, to the best of our knowledge a direct comparison of EEG frequency band power and pupil dilation data in more complex task situations has not been conducted up to now.

Thus, notwithstanding the limitations of the present work, our data showed for complex task materials and task situations simulating hypertext reading that both pupil dilation and EEG alpha band power overall are sensitive measures to assess the general load-situation during hyperlink-like selection processes, yet, alpha frequency band power might be the more specific measure for cognitive processes (i.e., EFs). The combined recording and analysis of eye-tracking and EEG frequency band data may be a promising methodological account to further study unconstrained, 'real-world' hypertext reading and link selection processes. Hence, this line of research may turn out to be highly relevant for the design and optimization of hypermedia learning environments. For example, future versions of Wikipedia articles may be optimized with regard to the kind and amount of hyperlinks so that additional load on readers' EFs is kept minimal, allowing to allocate more cognitive resources to processes of reading and comprehension. Especially as performance in EFs may be reduced in older adults [83] or impaired in certain populations (e.g., dyslexic or ADHD populations; [84,85]), assessment of load on EFs and the optimization of hypermedia environments with respect to amount and type of links therein causing this load might be valuable and necessary topics of future research. In this vein, individual differences research addressing, for example, the interaction between individuals' WM capacity or general executive function abilities and load during link selection processes might be carried out as well.

## Supporting Information

S1 Table Alpha Frequency Band Power and Pupil Dilation Data of Experiment 1 used for Statistical Analysis. (XLS)

S2 Table Alpha Frequency Band Power and Pupil Dilation Data of Experiment 2 used for Statistical Analysis. (XLS)

S3 Table Alpha Frequency Band Power and Pupil Dilation Data of Experiment 3 used for Statistical Analysis. (XLS)
